# Comparisons of complications between extended latissimus dorsi flap and latissimus dorsi flap in total breast reconstruction: A prospective cohort study

**DOI:** 10.1016/j.amsu.2020.05.046

**Published:** 2020-06-22

**Authors:** Prakasit Chirappapha, Panya Thaweepworadej, Kasamar Chitmetha, Chayanoot Rattadilok, Teerawut Rakchob, Thitipat Wattanakul, Panuwat Lertsithichai, Monchai Leesombatpaiboon, Nopawan Sanjaroensutikul

**Affiliations:** a Department of Surgery, Faculty of Medicine, Ramathibodi Hospital, Mahidol University, Thailand; b Department of Rehabilitation Medicine, Faculty of Medicine, Ramathibodi Hospital, Mahidol University, Thailand; c Department of Surgery, Bangkok Metropolitan Administration General Hospital, Bangkok, Thailand

**Keywords:** Extended latissimus dorsi, Latissimus dorsi, Shoulder function, Shoulder movement, Breast reconstruction

## Abstract

**Background:**

The latissimus dorsi (LD) flap is one of the most popular techniques in breast reconstruction. Although numerous studies have not shown functional impairment of the shoulder after surgery, other studies have reported significant functional impairment, especially after extended LD flap reconstruction. The present study compared functional deficit and shoulder movement between extended LD and LD flap reconstruction.

**Materials and methods:**

Between December 2015 and May 2018, this study enrolled 31 patients undergoing LD flap reconstruction. Data on patient demographics, operative details, morbidities, and degree of shoulder movement were collected. Outcomes were compared between the extended LD and LD flap groups.

**Results:**

Twenty-one women and 10 women underwent LD flap and extended LD flap reconstruction, respectively. The median patient age was 43 years. No demographic data differed between groups. Seroma, especially around the back incision, was the most common complication (90.5% in the LD flap and 90% in the extended LD group). Five patients in the LD flap group and one patient in the extended LD flap group showed decreased shoulder range of motion (ROM) at 6 months post-operation. Only one patient in the LD flap group showed impairment based on American Shoulder and Elbow Surgeons Shoulder Score (ASES). The results did not differ significantly between groups; however, the LD flap group showed faster functional recovery.

**Conclusion:**

LD flap reconstruction can be performed with a very low impact on shoulder function. We observed a slightly decreased ROM for both LD flap techniques, with no impact on functional outcome.

## Highlights

• After 6 months, 5 out of 16 patients in LD group and 1 out of 6 patients ELD group had decreasing of shoulder movement.
• Patients in ELD group needed more time to recover their movement compare with LD group (4 vs 1.5 months, respectively).
• According to our subjective outcome, only 1 patient in LD group had decreasing in her shoulder movement after 6 months.
• The median time to fully recovered of ASES score was only 1 month in both groups.
• All results were not statistically significant.

## Abbreviations

CI: confidence interval
DASH: Disabilities of the Arm, Shoulder and Hand
ELD: extended latissimus dorsi
IQR: interquartile range
LD: latissimus dorsi
ms-LD: muscle-sparing latissimus dorsi
NAC: nipple-areolar complex
NSM: nipple-sparing mastectomy
MRI: magnetic resonance imaging
ROM: range of motion
SD: standard deviation
SSM: skin-sparing mastectomy
STROCSS: Strengthening the Reporting of Cohort Studies in Surgery
TCTR: Thai Clinical Trials Registry
UN: United Nations

## 1 Introduction

According to recent data from The Global Cancer Observatory, more than 2 million new cases of breast cancer were diagnosed in 2018 [1]. Breast cancer is the most common cancer in Thai women and worldwide and its incidence is increasing. Surgical intervention plays a major role in breast cancer treatment, especially in early-stage cancer. Surgical management has evolved for more than a century from aggressive treatment to conservative approaches. Nowadays, aesthetic results are also an important factor to consider along with the oncological safety. Although breast-conserving surgery has shown comparable oncological results to more aggressive mastectomy techniques, mastectomy still has a role in some situations such as multicentricity, large tumor, post-radiated patients, and prophylaxis [2].

Latissimus dorsi (LD) myocutaneous flap is one of the most popular and feasible technique for breast reconstruction after mastectomy. It can be performed with low morbidity and does not require special instruments or microvascular surgery. It was first described in 1906 by Iginio Tansini and has gained popularity since that time [3]. Prosthetic implants are usually required to achieve adequate reconstructive volume due to the low volume of LD flaps. Hokin et al. first described the extended LD (ELD) flap technique in 1983, which harvested more parascapular and lumbar fat [4]. However, seroma, donor flap wound complication, and shoulder movement limitation were the major concerning complications with this technique. Previous studies reported donor-site seroma as well as delayed donor flap healing and necrosis in 25–70% and 14–21% of patients following ELD flap surgery [[5], [6], [7], [8]]. Although many studies have reported no functional impairment of the shoulder after LD flap surgery, others reported significant functional impairment in clinical practice, especially after LD flap reconstruction [9,10]. Therefore, combining LD flap with a prosthetic implant is increasingly popular to achieve adequate reconstructive breast volume and reduce donor-site morbidity. However, infection complications and capsular contracture are major concerns after prosthetic implant insertion [11]. Kim et al. [5] reported their experience on muscle-sparing LD flap technique with or without implant compared with ELD flap, observing less donor-site asymmetry and seroma (5.6% vs. 62.2%) for the muscle-sparing technique. At 4 weeks postoperatively, they found less limitation of shoulder movement for muscle-sparing techniques (25% vs. 75.7%). However, their study did not compare long-term results between these techniques. The present study compared operative complications, especially shoulder movement limitations and functional deficits, between LD and ELD flaps.

## 2 Material and methods

This prospective observational single-center cohort study enrolled 31 breast cancer patients undergoing LD flap reconstruction in our institute between December 2015 and May 2018. Patients older than 70 years of age, with a history of prior breast/shoulder surgery, and who underwent surgery for non-cancer indications were excluded from the study. Twenty-one and 10 women underwent immediate breast reconstruction with LD and ELD flap reconstruction, respectively. All patients received standard preoperative evaluations including clinical examinations, digital mammography, and breast ultrasonography. Magnetic resonance imaging (MRI) was not used routinely in our institute. Informed consent was obtained from all patients and the study was approved by the Institutional Review Board. Our work complied with the Strengthening the Reporting of Cohort Studies in Surgery (STROCSS) criteria [12]. The protocol is registered in the Thai Clinical Trials Registry (TCTR) which was approved by the United Nations (UN) (UIN TCTR20200106006).

**Fig. 1:**
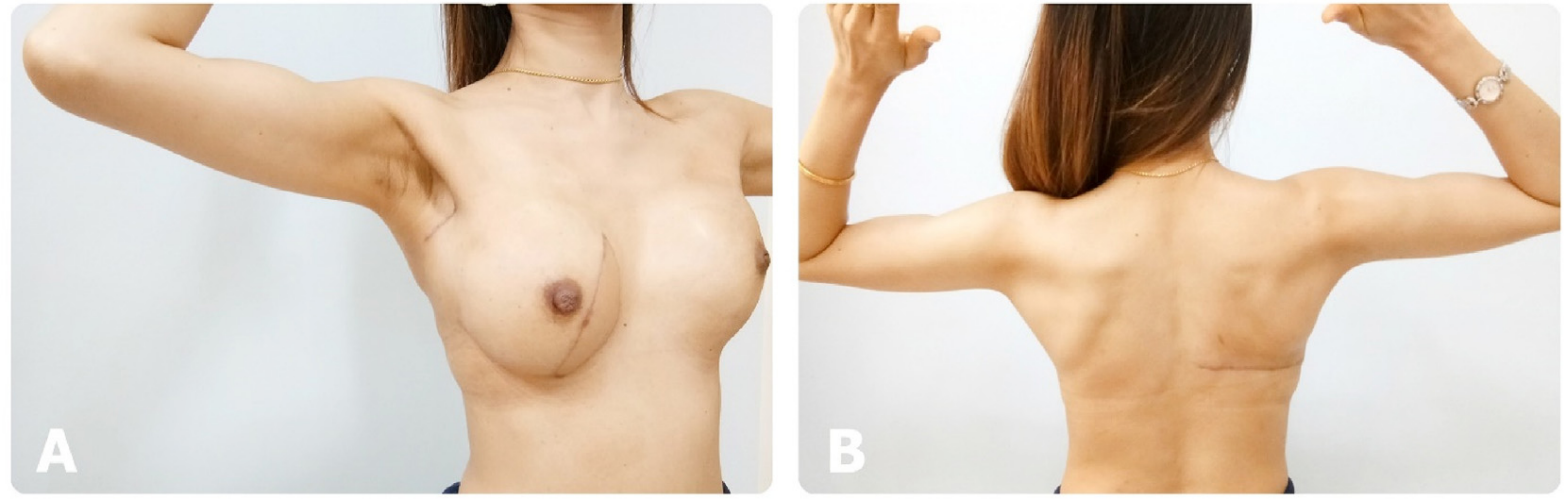
Shoulder movement 6 months after latissimus dorsi (LD) flap reconstruction. A) Anterior oblique view with external rotation. B) Posterior view with external rotation.

Data were collected on patient demographics, operative details, and postoperative morbidities. The specific complications assessed included surgical-site infection, donor or recipient flap necrosis, and seroma. Seroma formation was defined as a significant seroma with symptoms and seroma requiring intervention. All patients participated in the institutional rehabilitation program. Shoulder movement was evaluated pre- and postoperatively by comparisons to the non-operated side. We assessed shoulder movement according to the degree of motion in each direction as measured by goniometer and functional assessment based on the American Shoulder and Elbow Surgeons Shoulder Score (ASES). Some pictures are shown in Fig. 1. All assessments were performed by four surgical oncology surgeon fellows. Shoulder movement limitation was defined as a decreased degree of motion or functional score compared to the preoperative records.

### 2.1 Operative techniques

All operations were performed by a single surgeon with a team comprising fellows and residents of the Breast and Endocrinology unit in our institute. All patients received pre- and postoperative prophylaxis intravenous antibiotics. The operations proceeded as follows. Either skin-sparing mastectomy (SSM) or nipple-sparing mastectomy (NSM) was performed with the patient in a supine position. When performing NSM, we preferred superolateral radial incision to provide good exposure and low rate of necrotic complications [13]. The subareolar glandular tissue and all duct beneath nipple were cored out and sent for pathological examination by frozen section in all patients. The nipple-areolar complex (NAC) was excised if positive results were reported. Then position was changed to lateral decubitus with 90-degree upper arm abduction. The anterior border of LD muscle was marked and the tip of the flap was placed at the level of the brassiere line, at an estimated 10 cm below the axillary crease. An elliptical incision was performed over the LD muscle from the superior border of the flap to the identified LD muscle and descending branch of the thoracodorsal vessels. The posterior border of the flap was near the midline of the back, along the suprafascial layer of the LD muscle. In the LD flap group, a myocutaneous flap along the parascapular, scapular, and some part of the lumbar fat was harvested, while all parts of the LD muscle and lumbar fat were harvested in the ELD flap group. The thoracodorsal vessels, nerves, and tendinous insertion of the LD muscle were preserved. The pedicle flap was transferred to the breast pocket via a subcutaneous tunnel before the donor site was closed primarily. The patient was then placed in a supine position. A myocutaneous pedicle flap with or without prosthesis was placed in the pre-pectoral plane. The final decision in shape, size, and type of silicone prosthesis was made by physician preference. The subcutaneous tunnel around the pedicle was closed to prevent flap displacement. Two close suction drains were placed under the donor and recipient sites. Another suction drain was needed if axillary lymph node dissection was performed. After the content of each drain turned to clear serous fluid with a drainage volume of less than 30 mL per day for at least 2–3 days, the drain was removed.

The decision to administer adjuvant treatment was discussed with our multidisciplinary team. All patients were appointed to our outpatient department for weekly assessment in the first month and then monthly until 6 months postoperatively, then every 3 months for 2 years and 6 months for 5 years. Patients with follow-up times less than 6 months were excluded from the study.

### 2.2 Statistical analysis

All demographic data, tumor characteristics, complications, and oncological outcomes were presented with descriptive statistics. Means, standard deviations (SD), median, and interquartile ranges (IQRs) were used for continuous variables and frequencies and percentages for categorical variables. The primary outcome was shoulder movement limitation. The time-to-recovery of shoulder movement and postoperative complications were reported as secondary outcomes. Fisher's exact tests were used to identify associations between operative technique and each outcome. All statistical analysis was performed using STATA version 14.2.

## 3 Results

The average age of the 31 patients was 43 years old (range: 33–67 years). Most patients (74.19%) underwent an operation on their dominant side. Two of the 21 patients in the LD flap group received simple mastectomies due to phyllodes tumor and required LD flap for defect coverage. Twenty patients (64.52%) received breast reconstruction with prostheses. Patients in the ELD flap group had more advanced tumors and significantly more patients received adjuvant treatment compared to those in the LD flap group. The baseline patient characteristics are shown in Table 1.

**Table 1:** Baseline characteristics from 31 patients undergoing latissimus dorsi (LD) flap reconstruction.

	LD flap (N = 21) N (%) or median (IQR) or Mean ± SD	Extended LD flap (N = 10) N (%) or median (IQR) or Mean ± SD	P-value
Patient characteristics			
Age, yr	44 (10)	43 (10)	0.799
BMI, kg/m^2^	21.80 ± 2.55	23.22 ± 2.68	0.185
Menopausal status			0.532
Pre-menopause	3 (15)	0	
Post-menopause	17 (85)	10 (100)	
Dominant side	16 (76.19)	7 (70)	0.925
Operative data			
Breast			0.484
Mastectomy	2 (9.52)	0	
SSM	9 (42.86)	6 (60)	
NSM	10 (47.62)	4 (40)	
Axilla			0.073
SLNB	14 (66.67)	3 (30)	
ALND	5 (23.81)	6 (60)	
None	2 (9.52)	0	
Implant	17 (80.95)	3 (30)	*0.013*
Adjuvant treatment			
Adjuvant CMT	10 (47.62)	7 (70)	*0.023*
RT	5 (23.81)	5 (50)	*0.030*
Tumor characteristics			
Type			0.631
DCIS	2 (9.52)	1 (10)	
Invasive cancer	17 (80.95)	8 (80)	
Other	2 (9.52)	0	
Tumor size, cm	1.5 (2)	4.2 (2.1)	*0.014*
≤2	13 (61.90)	0	
2–5	6 (28.57)	7 (70)	
>5	2 (9.52)	2 (20)	
LN involvement			0.329
Negative	14 (66.67)	4 (40)	
1–3	3 (14.29)	3 (30)	
4–9	1 (4.76)	1 (10)	
>9	1 (4.76)	1 (10)	
Grade			0.962
I	1 (4.76)	1 (10)	
II	10 (47.62)	5 (50)	
III	7 (33.33)	2 (20)	
ER-positive	13 (61.90)	9 (90)	0.136
HER-2-positive	6 (28.57)	1 (10)	0.362
Ki-67			0.295
<20%	2 (9.52)	3 (30)	
≥20%	16 (76.19)	6 (60)	

Seroma, especially around the back incision, was the most common complication in our study (90.5% in LD flap and 90% in the ELD flap group). All were managed with 1–3 aspiration attempts. One of the 31 patients (3.23%) developed surgical site infection; the patient had undergone LD flap with a prosthesis and was managed conservatively with intravenous antibiotics. Two of the 31 patients in our series (6.45%) developed donor-site necrosis. The postoperative complications did not differ between groups (Table 2).

**Table 2:** Postoperative complications after 31 latissimus dorsi (LD) flap reconstructions.

	LD flap (N = 21) N (%) or mean ± SD	Extended LD flap (N = 10) N (%) or mean ± SD	P-value
Surgical site infection	1 (4.76)	0	0.999
Necrosis	1 (4.76)	1 (10)	0.999
Seroma			
Breast	0	0	
Axilla	1 (4.76)	1 (10)	0.999
Back	19 (90.48)	9 (90)	0.999
Others	0	0	

**Fig. 2:**
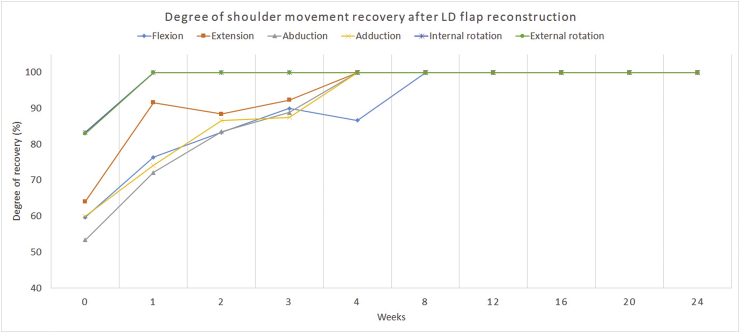
Average degree of shoulder movement recovery in each direction over time in 22 patients.

Only 22 patients completed the 6-month range-of-motion (ROM) examination. Five of 16 patients (31.25%) in the LD flap group and one of six patients (16.67%) in the ELD flap group had decreased shoulder movement after 6 months of operation. Flexion was the most common impaired direction after the operation, followed by extension and internal rotation (12.90, 9.68, and 9.68%, respectively). Patients in the ELD flap group required more time to recover shoulder movement compared to those in the LD flap group (4 vs. 1.5 months median time-to-recovery). However, the differences were not statistically significant. Data on shoulder ROM limitations are shown in Table 3. The average degree of shoulder movement recovery in each direction over time is shown in Fig. 2.

**Table 3:** Limitations of shoulder movement by direction in 22 patients undergoing different latissimus dorsi (LD) flap reconstructions techniques.

	LD flap (N = 16) N (%) or median (IQR)	Extended LD flap (N = 6) N (%) or median (IQR)	P-value
**Flexion**			
Limitation	3 (18.75)	1 (16.67)	0.999
Time to recovery (months)	1 (2.5)	4 (2)	0.098
**Extension**			
Limitation	3 (18.75)	0	0.549
Time to recovery (months)	0.5 (0.75)	1.12 (2.75)	0.449
**Abduction**			
Limitation	1 (6.25)	0	0.999
Time to recovery (months)	1 (2.25)	2.50 (1)	0.154
**Adduction**			
Limitation	0	0	
Time to recovery (months)	0.62 (2.37)	2 (3.25)	0.167
**Internal rotation**			
Limitation	2 (12.5)	1	0.999
Time to recovery (months)	0.25 (0.75)	2 (2.75)	0.285
**External rotation**			
Limitation	2 (12.5)	0	0.999
Time to recovery (months)	0.63 (1.75)	0.88 (1.75)	0.646

**Fig. 3:**
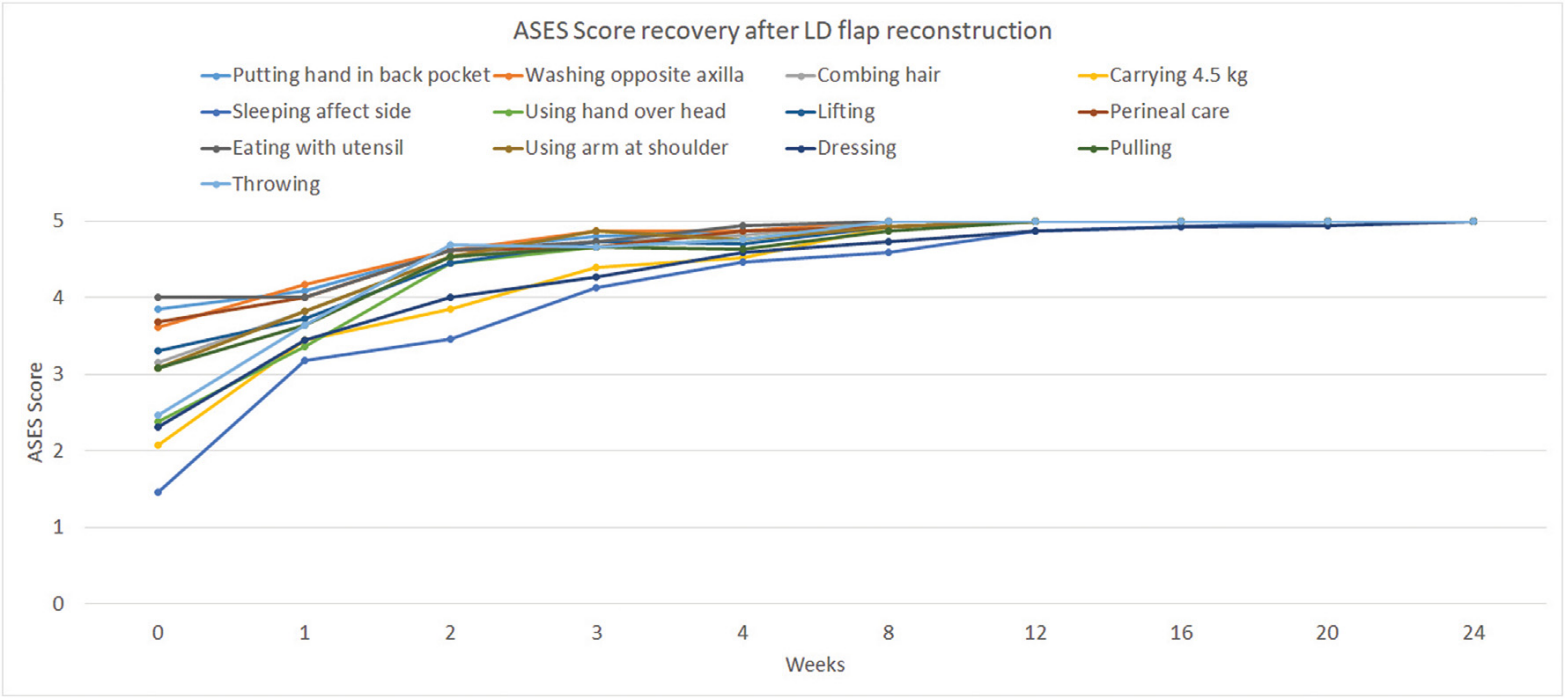
Mean American Shoulder and Elbow Surgeons Shoulder Score (ASES) recovery in each activity over time in 25 patients.

Evaluation of ASES in 25 patients with complete 6-month examinations showed decreased shoulder movement in one female patient in the LD flap group. The median time to full recovery of all functions in the ASES was only 1 month in both groups. Comparisons of each question in ASES showed significantly longer time-to-recovery for the tasks of “wash your back/do up bra” and “throw a ball overhand” in the ELD flap group. ASES limitation data are shown in Table 4. The mean ASES score recovery in each activity over time is shown in Fig. 3.

**Table 4:** @Limitations of shoulder movement by activities in 25 patients undergoing different latissimus dorsi (LD) flap reconstructions techniques.

	LD flap (N = 16) N (%) or median (IQR)	Extended LD flap (N = 9) N (%) or median (IQR)	P-value
**Putting hand in back pocket**			
Limitation	0	0	
Time to recovery (months)	0 (0.75)	1 (0.25)	*0.022*
**Washing opposite axilla**			
Limitation	0	0	
Time to recovery (months)	0 (0.75)	1 (0.25)	*0.022*
**Combing hair**			
Limitation	0	0	
Time to recovery (months)	0.625 (1.75)	1 (0.25)	0.159
**Carrying 4.5 kg**			
Limitation	0	0	
Time to recovery (months)	0.75 (1.75)	1 (1.25)	0.453
**Sleeping of the affected side**			
Limitation	0	0	
Time to recovery (months)	1 (2.25)	1 (0.25)	0.333
**Using hand over head**			
Limitation	0	0	
Time to recovery (months)	0.75 (1.5)	1 (0.25)	0.390
**Lifting**			
Limitation	0	0	
Time to recovery (months)	0.5 (0.75)	1 (0.25)	*0.036*
**Perineal care**			
Limitation	0	0	
Time to recovery (months)	0.5 (0.75)	1 (0.25)	0.054
**Eating with utensil**			
Limitation	0	0	
Time to recovery (months)	0.25 (0.75)	1 (0.50)	0.080
**Using arm at shoulder**			
Limitation	0	0	
Time to recovery (months)	0.75 (0.5)	1 (0.25)	0.223
**Dressing**			
Limitation	1 (6.25)	0	0.999
Time to recovery (month)	0.875 (2.37)	1 (1.25)	0.445
**Pulling**			
Limitation	0	0	
Time to recovery (months)	0.5 (1)	1 (0.25)	0.141
**Throwing**			
Limitation	0	0	
Time to recovery (months)	0.5 (0.5)	1 (0.25)	*0.041*

## 4 Discussion

The LD plays a major role in shoulder movement and stability. Many patients are concerned about functional impairment of their shoulder after reconstruction, especially following ELD flap reconstruction. Most previous studies were retrospective in nature and provided limited data on ELD flap reconstruction. These studies reported varying results and measurement techniques. Clough et al. [14] reported 40% subjective overall discomfort after ELD flap harvest in 30 patients. They also performed an objective assessment by physical examination of three activities and observed limitations in 47% of their patients. The mean evaluation time was 19 months postoperatively. Conversely, recent studies reported no significant impairment after long-term follow-up. A prospective series by Eyjolfsdottir et al. [15] reported significantly reduced shoulder ROM at 1 and 6 months postoperatively in 15 patients who underwent ELD flap operation, all of whom achieved full range of shoulder movement after 12 months. The authors also observed found slightly impaired spine mobility and muscle strength after ELD flap operation. However, their study lacked a comparator group. Glassey et al. [16] compared eight patients who underwent ELD flap reconstruction to 14 who received traditional LD flap reconstruction. Both groups also reported limitations in shoulder ROM and strength at 6 weeks postoperatively but had fully recovered after 1 year. Patients in the ELD subgroup only showed poorer recovery in disability score by Disabilities of the Arm, Shoulder and Hand (DASH) questionnaire compared to that in the traditional LD group. Another study from Kim et al. [5] compared objective outcomes based on shoulder ROM between 37 patients who underwent ELD flap surgery and 36 who underwent muscle-sparing LD (ms-LD) flap harvest. They reported significantly more limitations in the ELD patients at 1 month postoperatively (75.7% vs. 25% in the ms-LD group, *p* = 0.0001). However, the shoulder function recovered and no significant difference was observed after 6 months (8% vs. 0% in ELD and ms-LD, respectively, *p* = 0.081).

We evaluated shoulder function based on both subjective and objective outcomes. Objective evaluation allows more accurate comparisons of ROM function between pre- and postoperative values. However, subjective outcomes have more clinical impact. In our study, six of 22 (27.27%) patients had some degree of shoulder ROM limitation after 6 months. All of them had restrictions in only one or two directions, with an average motion decrease of 16.18°. In our study, traditional LD flap did not show better ROM recovery compared to that for ELD flap reconstruction. It is hard to compare results between studies due to numerous confounding factors and differences in measurement techniques. Many factors may affect the functional outcome of the shoulder, including dominant-side operation, radiotherapy, or surgical technique. Previous studies reported shoulder movement restriction following mastectomy alone [17]. Unlike other studies, we performed LD flap reconstruction in breast cancer patients after mastectomy. Fifteen of the 22 patients (68.18%) in our study received adjuvant treatment, 36.36% of whom received adjuvant radiotherapy. Five of six patients (83.33%) with shoulder movement restriction underwent the operation on their dominant side.

The limitations of our study are the low data volume and missing data (nine and six patients had incomplete ROM evaluation and missing subjective ASES data, respectively). The study was underpowered to make conclusions regarding the differences in each reconstruction technique. However, our data support the limited shoulder movement in the early postoperative period that quickly recovered after the first 8 weeks (Fig. 2, Fig. 3). After that, while several patients had remaining shoulder movement limitation the clinical impact was very low. ELD flap reconstruction can be done with low morbidity and no difference in functional outcome compared to those for the traditional LD flap. More prospective data are needed to determine and compare the impacts on shoulder function between each type of reconstruction.

## 5 Conclusion

LD myocutaneous flap can be performed with a very low impact on shoulder function. We observed a slightly decreased ROM for both LD flap techniques but no impact on functional outcome in ASES. The traditional LD flap showed faster functional recovery compared to that for the ELD flap technique.

## Ethical approval

Committee on Human Rights Related to Research Involving Human Subjects.

Faculty of Medicine Ramathibodi Hospital, Mahidol University.

Protocol Number: ID 06-61-48.

## Sources of funding

No grants or financial support were received by any of the authors in relation to this study or to the writing of this article.

## Author contribution

1. Assoc. Prof. Prakasit Chirappapha
  • Department of Surgery, Faculty of Medicine Ramathibodi Hospital, Mahidol University, Bangkok, Thailand
  • Writing manuscript and interpretation of data
2. Dr. Panya Thaweepworadej
  • Department of Surgery, Bangkok Metropolitan Administration General Hospital, Bangkok, Thailand
  • Department of Surgery, Faculty of Medicine Ramathibodi Hospital, Mahidol University, Bangkok, Thailand
  • Correspondent, interpretation of data and analysis
3. Dr. Kasamar Chitmetha
  • Department of Surgery, Faculty of Medicine Ramathibodi Hospital, Mahidol University, Bangkok, Thailand
  • Acquisition of data
4. Dr. Chayanoot Rattadilok
  ∙ Department of Surgery, Faculty of Medicine Ramathibodi Hospital, Mahidol University, Bangkok, Thailand
  ∙ Acquisition of data
5. Dr. Teerawut Rakchob
  ∙ Department of Surgery, Faculty of Medicine Ramathibodi Hospital, Mahidol University, Bangkok, Thailand
  ∙ Acquisition of data
6. Dr. Thitipat Wattanakul
  • Department of Surgery, Faculty of Medicine Ramathibodi Hospital, Mahidol University, Bangkok, Thailand
  • Acquisition of data
7. Assoc. Prof. Panuwat Lertsithichai
  • Department of Surgery, Faculty of Medicine Ramathibodi Hospital, Mahidol University, Bangkok, Thailand
  • Acquisition of data
8. Dr. Monchai Leesombatpaiboon
  • Department of Surgery, Faculty of Medicine Ramathibodi Hospital, Mahidol University, Bangkok, Thailand
  • Acquisition of data
9. Dr. Nopawan Sanjaroensutikul
  • Department of Rehabilitation Medicine, Faculty of Medicine Ramathibodi Hospital, Mahidol University, Bangkok, Thailand
  • Physical Medicine and Rehabilitation specialist, acquisition of data

## Research registry number

The protocol had been registered at Thai Clinical Trials Registry (TCTR). The identification number is TCTR20200106006.

http://www.clinicaltrials.in.th/index.php?tp=regtrials&menu=trialsearch&smenu=fulltext&task=search&task2=view1&id=5620.

## Guarantor

Dr. Panya Thaweepworadej (Corresponding author).

Department of Surgery, Bangkok Metropolitan Administration General Hospital, Bangkok, Thailand.

+662-220-8000

thaweep@klanghospital.go.th.

## Consent

Written informed consent was obtained from all patients for the publication of this case report and accompanying images. Copies of the written consents are available for review by the Editor-in-Chief of this journal upon request.

## Provenance and peer review

Not commissioned, externally peer reviewed.

## Declaration of competing interest

All authors have no any financial and personal relationships with other people or organization that could inappropriately influence (bias) their work.
